# Prediction of Response to Immunotherapy in Hepatocellular Carcinoma: Where Do We Stand?

**DOI:** 10.3390/cancers18193167

**Published:** 2026-10-01

**Authors:** Mariana Verdelho Machado

**Affiliations:** 1 Clínica Universitária de Gastrenterologia, Faculdade de Medicina, Universidade de Lisboa, 1649-028 Lisboa, Portugal; mverdelhomachado@gmail.com or mariana.verdelho.machado@fundacaochampalimaud.pt; 2 Unidade de Oncologia Digestiva, Fundação Champalimaud, 1400-038 Lisboa, Portugal; 3 Serviço de Gastrenterologia, Hospital de Vila Franca de Xira, 2600-009 Vila Franca de Xira, Portugal

**Keywords:** biomarkers, hepatocellular carcinoma, immune checkpoint inhibitors-based therapy

## Abstract

Worldwide, liver cancer is the seventh most common cancer and the third leading cause of cancer-related mortality, reflecting its dismal prognosis, with only approximately 20% of patients alive 5 years after diagnosis. At least 50% of patients ultimately require systemic treatment. In 2020, the systemic treatment landscape changed dramatically with the introduction of immune checkpoint inhibitor (ICI)-based therapies, which have demonstrated improved overall survival and longer preservation of quality of life compared with tyrosine kinase inhibitor (TKI)-based approaches. However, fewer than one third of patients achieve objective response, 10–25% experience severe immune-related adverse events, and ICI-based treatments represent a substantial economic burden for health systems. Importantly, robust and clinically validated predictive biomarkers of response to ICI remain an unmet need. This review summarizes current efforts to identify predictive biomarkers of ICI response in hepatocellular carcinoma (HCC), integrating clinical, pathological, molecular, microbiome, and imaging approaches, and discusses how these developments may shape the future of treatment selection.

## Simple Summary

The million-dollar question regarding immune checkpoint inhibitor (ICI)-based therapies for patients with hepatocellular carcinoma (HCC) is how to predict who will benefit from treatment and who should be considered for alternative approaches. Although responders can achieve prolonged survival and, in some cases, experience downstaging or stage migration that enables potentially curative treatments that were previously not feasible, fewer than one-third of patients achieve an objective response. Moreover, disease progression after first-line treatment may limit subsequent therapeutic options. Currently, several prognostic biomarkers provide information about overall prognosis in patients with HCC, and promising simple on-treatment biomarkers are emerging. However, unlike in several other solid tumors, there are currently no established biomarkers or tools that can reliably predict response to immunotherapy in HCC. Research addressing this critical question is rapidly evolving, offering hope for the development of clinically useful predictive tools in the near future. This review provides a comprehensive overview of the current state of the art in biomarkers for predicting response to ICI-based therapies for patients with HCC.

## 1. Introduction

Globally, liver cancer ranked seventh in terms of cancer incidence and third as a cause of cancer-related mortality in 2024 [1]. Hepatocellular carcinoma (HCC) has a high mortality-to-incidence ratio, approaching 1, which underscores its poor prognosis. Indeed, the 5-year overall survival of patients with HCC is approximately 20% [2], reflecting the aggressive biology of the disease and the limited efficacy of available therapeutic options.

The first systemic therapy for HCC was only approved in 2007–2008, after the SHARP trial demonstrated a survival benefit of 3 months with sorafenib compared with placebo, and an objective response rate (ORR) below 5% [3]. The introduction of other tyrosine kinase inhibitors (TKIs), particularly lenvatinib, has provided additional treatment options, with the REFLECT trial demonstrating non-inferiority of lenvatinib to sorafenib in overall survival (OS) [4].

The treatment landscape changed substantially with the emergence of immune checkpoint inhibitor (ICI)-based therapies in 2020. Several phase III trials (Table 1) have demonstrated that ICIs provide survival benefits, with median survival 2.5–9 months longer as compared to sorafenib, ORR of approximately 20–36%, and complete responses in approximately 1–8% of patients [5,6,7,8,9,10,11]. Importantly, a subset of patients derive particularly durable benefits, with up to 20% of patients being alive at 5 years [8]. Furthermore, ICI-based therapies can provide better preservation of health-related quality of life and delay deterioration compared with sorafenib [12,13,14].

Currently, several ICI-based regimens are recommended as first-line treatments for patients with advanced HCC and preserved liver function. These include atezolizumab plus bevacizumab (AB), durvalumab plus tremelimumab (DT) administered according to the STRIDE regimen, and nivolumab plus ipilimumab (NI) [15,16,17]. In addition, Chinese guidelines recommend camrelizumab plus rivoceranib and sintilimab plus the bevacizumab biosimilar IBI305 based on phase III trials conducted predominantly in Asian populations with a high prevalence of hepatitis B virus-associated HCC [18].

Despite these major advantages, one-third of patients are primary non-responders to ICI-based therapy, and hyperprogressive disease has been reported in approximately 10% of patients in some HCC cohorts [19]. Moreover, the efficacy of first-line treatment has important implications for subsequent treatment opportunities. Disease progression may be accompanied by deterioration in hepatic function and performance status, potentially precluding further systemic therapy. Indeed, real-world studies have shown that only 25% to 40% of patients are able to receive second-line therapy after discontinuation of ICIs [20].

ICI-based therapies are also associated with clinically relevant immune-related adverse events (irAE). The risk is particularly high for regimens incorporating dual immune checkpoint blockage, with high doses of corticosteroids required in approximately 10% for AB, 20% for DT, and up to 30% for NI [14,21,22]. Finally, ICI-based regimens represent costly therapies and may impose a substantial economic burden on healthcare systems.

Taken together, these considerations highlight an important unmet clinical need: the ability to identify, before treatment initiation, which patients are most likely to derive substantial and durable benefits from ICI-based therapy and which patients may be better served by alternative strategies, such as TKI-based treatment. Therefore, a reliable predictive biomarker could not only improve treatment efficacy but also reduce unnecessary toxicity, preserve subsequent treatment opportunities, and improve the cost-effectiveness of systemic therapy for HCC.

In other solid tumors, several predictive biomarkers have been incorporated into clinical practice and treatment algorithms, including programmed death ligand-1 (PD-L1) expression, microsatellite instability-high/mismatch repair deficiency (MSI-H/dMMR), and tumor mutational burden-high (TMB-H) [23]. For example, PD-L1 expression is routinely used to guide immunotherapy selection in lung [24] and head and neck cancers [25], while MSI-H/dMMR status is an established biomarker for treatment selection in colorectal cancer [26]. However, these established biomarkers have limited applicability to HCC, with no biomarker yet approved for selecting patients for ICI-based therapy. This review summarizes the current evidence on biomarkers for predicting response to ICI-based treatment in HCC, with particular emphasis on emerging approaches and advances that may enable their integration into clinical practice in the near future.

## 2. Mechanisms of Resistance to ICI-Based Therapies

The immune system plays a dual role in cancer, with the capacity to both promote tumorigenesis and mount effective antitumor responses. Through immunosurveillance, immune cells can recognize and eliminate transformed cells by sensing tumor-associated antigens and other signals of cellular stress. However, tumors can evade immune-mediated elimination through multiple mechanisms, including the exploitation of inhibitory immune checkpoint pathways that restrain T cell activation and function. ICI-based therapies aim to restore antitumor immunity by disrupting these inhibitory pathways.

T cell activation requires two signals: antigen-specific signaling through the T cell receptor (TCR) and costimulatory signals. The TCR recognizes its specific peptide antigen presented by antigen-presenting cells (APCs). The costimulatory receptor CD28 interacts with the B7 family members CD80 and CD86 on APCs [27]. The activation of T cells can be regulated by inhibitory immune checkpoint pathways. Cytotoxic T-lymphocyte-associated protein-4 (CTLA-4), expressed on activated T cells and constitutively on regulatory T cells (Tregs), competes with CD28 for CD80 and CD86 and thereby attenuates costimulatory signaling and T cell priming [28]. CTLA-4 also contributes directly to the immunosuppressive functions of Tregs. Another inhibitory pathway involves programmed death protein-1 (PD-1), which is expressed on activated T cells and other immune cell populations (e.g., B cells and NK cells). Engagement of PD-1 with its ligand PD-L1 delivers inhibitory signals that reduce T cell proliferation and function, and in the setting of persistent antigen stimulation, can contribute to dysfunctional or exhausted T cell states. Several tumors, including HCC, can exploit PD-L1 expression as a mechanism of immune evasion [28].

ICIs are monoclonal antibodies that block inhibitory immune checkpoint interactions, targeting PD-1 (pembrolizumab, nivolumab, camrelizumab, and sintilimab), PD-L1 (atezolizumab and durvalumab), and CTLA-4 (tremelimumab and ipilimumab), and hence can enhance or reinvigorate antitumor T cell responses and promote immune-mediated tumor-cell elimination.

Nevertheless, immune evasion can occur through multiple mechanisms beyond immune checkpoint signaling. These include antigen loss and immunoediting, production of immunosuppressive cytokines, and recruitment or reprogramming of immune cells toward immunosuppressive and pro-tumorigenic phenotypes. Among these Tregs, tumor-associated macrophages (TAMs), tumor-associated neutrophils (TANs), and myeloid-derived suppressor cells (MDSCs) are important components of the immunosuppressive tumor microenvironment [28,29].

Tregs are a specialized subset of CD4+T cells that are essential for maintaining self-tolerance and preventing excessive immune activation; however, their immunosuppressive activity can impair effective antitumor immunity. Tregs suppress APC function and effector T cell activation through several mechanisms, including secretion of immunosuppressive cytokines such as IL-10 and TGF-β, sequestration of IL-2, competition for costimulatory signals through high-level expression of CTLA-4, and direct cytotoxicity toward effector T cells, NK cells, and dendritic cells [30].

TAMs can acquire immunosuppressive and tumor-promoting M2-like phenotypes, playing a central role in immune evasion. TAMs suppress T- and NK-cell functions through cytokines such as IL-10 and TGF-β, reshape the tumor microenvironment through recruitment of Tregs, impair dendritic-cell maturation and antigen presentation, and remodel the extracellular matrix and tumor vasculature through mediators such as VEGF [31].

TANs can also acquire a pro-tumorigenic “N2” phenotype that suppresses T and NK cell responses through the release of reactive oxygen species, proteases, arginase, and other mediators, and can promote the recruitment of Tregs. Furthermore, TANs contribute to tumor progression by reshaping the tumor microenvironment through angiogenesis, extracellular matrix remodeling, and can form neutrophil extracellular traps (NETs) that coat tumor cells, facilitating tumor-cell survival, immune evasion, invasion, and metastatic dissemination [32].

MDSCs are a heterogeneous population of immature pathologically activated myeloid cells that accumulate in many cancers and act as central drivers of immune evasion. They can blunt innate and adaptive antitumor immunity through multiple mechanisms. For example, amino acid depletion and metabolic starvation of T cells, production of reactive oxygen and nitrogen species, induction of T cell apoptosis, and recruitment of Tregs and polarization of TAMs toward an M2-like phenotype [33].

More recently, cancer-associated fibroblasts (CAFs) have emerged as important mediators of immune evasion within the tumor microenvironment. CAFs can promote immunosuppression through the secretion of cytokines and chemokines that impair effector T cell function and recruit or modulate immunosuppressive populations, including Tregs and TAMs. In addition, CAF-mediated extracellular-matrix remodeling can generate physical and biochemical barriers to the infiltration of effector lymphocytes, while promoting tumor cell invasion and metastatic dissemination [34].

**Figure 1:**
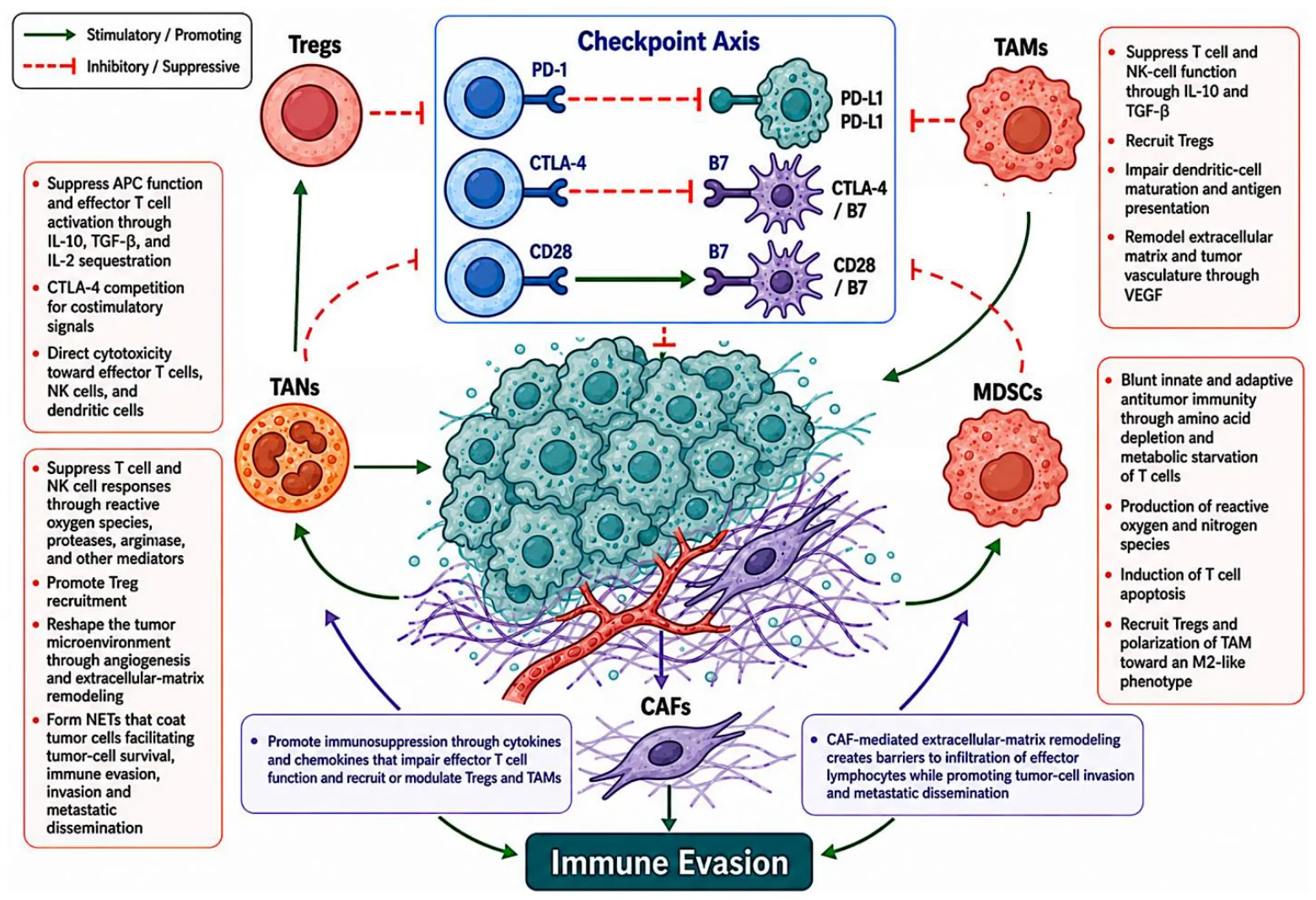
Immune checkpoint pathway and tumor microenvironment resistance. CAF, cancer-associated fibroblasts; MDSC, myeloid-derived suppressor cells; TAM, tumor-associated macrophages; TAN, tumor-associated neutrophils; Treg, regulatory T cells. [Figure designed with the help of AI, Paperpal Illustrate BETA].

Collectively, these mechanisms and cell players may contribute to resistance to ICI-based therapies [35,36] (Figure 1).

## 3. Biomarkers to Predict Response to ICI-Based Therapies

Biomarkers can be classified as prognostic, predictive, and on-treatment biomarkers [37,38]. Prognostic biomarkers provide information about a patient’s outcome independently of treatment, whereas predictive biomarkers identify patients who are likely to benefit from a specific treatment. In contrast, on-treatment biomarkers provide information regarding treatment response early after its initiation, potentially enabling timely reconsideration of the treatment strategy. Although this categorization is not always straightforward, as some biomarkers may have both prognostic and predictive value, most baseline parameters used to assess patient suitability for ICI-based therapies in HCC are primarily prognostic or reflect disease severity, such as liver function and baseline tumor markers including α-fetoprotein (AFP). Conversely, changes observed after treatment initiation, such as AFP kinetics and early radiological response, can provide an early assessment of treatment efficacy and may help guide subsequent treatment decisions. A substantial body of research is currently focused on the identification of predictive biomarkers, leveraging sophisticated approaches including genomics, bulk or single-cell and spatial transcriptomics, multiplexed imaging, radiomics, and artificial intelligence (AI). These studies have provided invaluable insights into the biological mechanisms underlying resistance to ICI-based therapies and pave the way for the development of new clinically applicable predictive tools (Table 2).

### 3.1. Clinical Parameters

Regarding sex, the difference in response to ICI-based therapies in HCC remains inconclusive. Although most studies suggest that male patients derive greater benefit [126,127,128], meta-analyses and real-world studies have not demonstrated significant differences in treatment outcomes according to sex [126,129]. Moreover, the available evidence is characterized by substantial methodological limitations and clinical heterogeneity.

Age does not appear to significantly influence the response to ICI-based therapies in HCC [129]. Elderly patients, including those older than 65 and even 75 years, appear to derive similar clinical benefits compared with younger patients [130,131].

A “body mass index (BMI) paradox” has been described, whereby overweight or obese patients receiving ICI-based therapies appear to have higher ORR and DCR, as well as longer PFS and OS [132,133]. Proposed biological mechanisms to explain this association include the effects of obesity-related metabolic and inflammatory processes, and in particular, the potential role of leptin in modulating T cell function and PD-1 expression [134]. However, the available evidence is largely derived from retrospective studies and may be affected by confounding factors, including undernutrition and sarcopenia [135,136]. Sarcopenia has been associated with lower ORR, with shorter PFS and OS [137,138], even though its clinical value appears to be predominantly prognostic rather than predictive of ICI benefit [139,140]. Interestingly, changes in body composition during treatment may provide additional information. In one retrospective study, patients experiencing rapid weight loss had approximately a four-fold higher risk of mortality than those with stable or increasing body weight [141]. Similarly, the development of sarcopenia during treatment was more consistently associated with shorter PFS in patients receiving ICI-based therapies than the presence of pre-treatment sarcopenia [142].

A concern regarding the potential influence of liver disease etiology on the efficacy of ICI-based therapies was first raised by a preclinical study by Pfister et al. [143]. This study suggested that patients with metabolic dysfunction-associated steatotic liver disease (MASLD)-associated HCC might be intrinsically less responsive to ICI-based therapy due to progressive accumulation of exhausted CD8+PD1+T cells, with impaired antitumor activity. The authors then performed a meta-analysis of three phase III trials in more than 1600 HCC patients and reported that, unlike in viral-associated HCC, ICI-based therapies did not improve survival in non-viral patients. Consistent with these findings, the main clinical trials, including CheckMate 459 with nivolumab [14], IMbrave 150 with AB [5,6], KEYNOTE 240 with pembrolizumab [144], and CARES-310 with camrelizumab plus rivoceranib [10,145], did not demonstrate a survival benefit in non-viral patients. In contrast, the HIMALAYA trial showed the efficacy of DT also in non-viral HCC [7]. Hepatitis C virus-associated HCC might also differ in its response to ICI-based therapy. In KEYNOTE 240 [144], HIMALAYA [7], and CARES-310 [10,145] no significant OS benefit was observed in the hepatitis C subgroup. However, these findings should be interpreted cautiously, as non-viral etiologies were underrepresented in clinical trials and were not stratified between MASLD and alcohol-associated liver disease. Subsequent meta-analyses of clinical trials have generally demonstrated a survival benefit also in non-viral etiologies, although some analyses suggest a greater magnitude of benefit in viral-associated HCC [146,147,148,149]. Among ICI-based regimens, DT may provide a greater benefit in non-viral-associated HCC [146]. Meta-analyses also suggest comparable efficacy in hepatitis B- or C-virus-associated HCC [146]. Importantly, real-world studies have not consistently demonstrated differences in response to ICI-based therapy according to the etiology of liver disease [150,151,152]. Accordingly, current international guidelines do not recommend different systemic treatment regimens based on HCC etiology [15,16,17].

Liver function, assessed by the Albumin-Bilirubin (ALBI) or CPT score, reflects hepatic functional reserve and prognosis. However, liver function impairment may also be associated with altered immunity, potentially reducing the efficacy of ICI-based therapies. Indeed, in a phase II trial of 16 patients treated with nivolumab plus sorafenib, patients with CPT class B, compared with those with class A, had increased levels of angiogenic and immunoregulatory mediators, including TGF-β, together with a distinct immune-cell profile characterized by suppressive monocytes and reduced effector T cell activity [153]. Current guidelines recommend systemic treatment primarily for patients with preserved liver function, and pivotal clinical trials have predominantly enrolled patients from CPT class A. Consequently, real-world studies are particularly relevant for evaluating the potential benefit of ICI-based therapies in patients with CPT class B. Retrospective studies and meta-analyses consistently demonstrated shorter OS in CPT class B compared with class A, with median OS decreasing from approximately 17 to 7 months [39,40,41,42,43,44,45]. The ORR appears to be similar or only modestly lower in CPT B patients, but CPT B patients generally have shorter treatment exposure and inferior PFS [39,40,41,42,43,44,45]. Indeed, the reduction in OS appears to be only partly attributable to diminished oncologic efficacy and is largely driven by liver disease progression, with approximately two-thirds of deaths attributed to hepatic decompensation in some cohorts, suggesting a predominantly prognostic rather than predictive value [42,46]. Regarding the safety profile of ICI-based therapy in CPT B patients, findings are not entirely consistent [42,43]; however, most studies do not indicate an increased risk of severe adverse events compared with CPT A patients [39,40,44,153]. More importantly, retrospective studies including CPT B HCC patients treated with either ICI-based regimens or sorafenib or standard-of-care suggest that ICI-based therapies may be associated with an approximately 50% reduction in the risk of death and an improvement in median OS from approximately 4 to 8 months [44,47]. These findings support the concept that selected patients with CPT B can still derive clinically meaningful benefit from ICI-based therapies. Notably, CPT class B encompasses a broad spectrum of hepatic dysfunction, ranging from minor laboratory abnormalities to persistent hepatic decompensation. Survival progressively decreases with each point increase in CPT score from 5 to 9 [44,154]. Accordingly, patients with CPT 7–8 points may still benefit from ICI-based therapy, and 10–30% may even experience an improvement in liver function, particularly when an objective response is achieved. Marked tumor shrinkage (>50%) may be particularly relevant, as the tumor burden itself can contribute to portal hypertension and deterioration of hepatic function [46,155,156]. Conversely, in patients with CPT 9 or higher or persistent hepatic decompensation, prognosis is extremely poor, and HCC-directed systemic therapy is unlikely to provide meaningful benefit [43,157]. Among patients receiving ICI-based therapy, pre-existing or treatment-emergent ascites, particularly high-volume ascites, appears to be a strong predictor of mortality [158,159,160]. Beyond CPT, the ALBI and modified ALBI (mALBI) grades may provide a more objective and refined assessment of hepatic reserve, using only two objective continuous variables: bilirubin and albumin. Indeed, ALBI and mALBI appear to more accurately stratify prognosis than CPT in patients receiving ICI-based therapies [48,49,50]. They also provide additional prognostic discrimination within both CPT class A [51] and B [45], with particularly poor survival observed in patients with (m)ALBI 2(b) or 3 [52,53]. Overall, these data suggest that treatment decisions in CPT B should incorporate the severity and trajectory of hepatic dysfunction, the presence and extent of decompensation, and ALBI/mALBI grade, together with the likelihood of achieving meaningful tumor response.

The radiological phenotype of HCC tumors also carries prognostic significance, with infiltrative tumors being associated with shorter median PFS and OS [161,162]. These tumors are characterized by increased expression of proliferative and cell cycle-related markers, together with enhanced TGF-β signaling and an immunosuppressive tumor microenvironment including higher Treg-cell signatures, which may contribute to their aggressive clinical behavior and poorer outcomes [162].

Radiological monitoring and assessment of treatment response at 6–8 weeks are critical for clinical decision-making. In HCC, the modified RECIST (mRECIST)-1.1 criteria are recommended for response assessment, as they account for the total size of lesions and viable arterial-enhancing tumor components [38]. Indeed, in HCC, mRECIST-1.1 has been associated with improved prognostic discrimination compared with RECIST-1.1, although supporting meta-analytic evidence is predominantly derived from patients receiving TKI rather than ICI-based therapies [79]. In ICI-treated HCC, small retrospective studies have not demonstrated a consistent superiority of mRECIST-1.1 over RECIST-1.1 [163], with mRECIST-1.1 detecting more responses, but RECIST-1.1 having similar prognostic discriminative capacity [164]. In the ICI-therapy setting, iRECIST was developed to account for atypical response patterns such as pseudoprogression; however, comparative studies in HCC have not demonstrated an advantage of iRECIST over mRECIST-1.1 [163]. Moreover, pseudoprogression appears to be uncommon in HCC patients treated with ICI-based therapies [38].

Lastly, the occurrence of IRAE appears to be associated with improved outcomes in patients receiving ICI-based therapies, suggesting their potential role as on-treatment biomarkers of treatment benefit. Recent meta-analyses have reported an approximately 2-fold higher odds of achieving ORR and 3-fold DCR, together with an approximately 38% lower risk of progression and, in some analyses, a 24–34% reduction in mortality. These associations appear to be particularly robust in low-grade (1–2) IRAE [22,165,166,167,168]. The effect is not uniform across IRAE types, with dermatologic and endocrine events showing the most consistent association with improved outcomes [22,165,168]. Proposed mechanisms include antigenic mimicry and expansion of shared CD8+ effector memory T cell populations, potentially reflecting a more robust systemic immune response to ICI therapy [165]. Importantly, corticosteroid treatment for IRAE did not appear to compromise outcomes [22,169], in contrast with corticosteroid use for palliation of cancer-related symptoms, which has been associated with lower OS, although this association may be confounded by the poor prognosis and disease burden of the latter [169].

### 3.2. Blood Tests and Oncofetal Tumor Markers

AFP is an oncofetal protein whose expression declines dramatically after birth but can be re-expressed and secreted by poorly differentiated malignant HCC cells [170]. Elevated serum AFP levels in HCC patients are associated with advanced disease and poorer outcomes, including shorter OS following locoregional therapy, surgery, or systemic TKI treatment [171,172,173]. AFP also has a predictive role in the selection of patients for ramucirumab, as clinical benefit was demonstrated only in patients with baseline AFP ≥400 ng/mL [174]. In contrast, the role of baseline AFP in predicting response to ICI-based therapies appears limited. AFP primarily reflects tumor burden, but it may also contribute mechanistically to immune escape [175]. Preclinical studies suggest that AFP may exert immunosuppressive effects through several mechanisms, including induction of apoptosis and inhibition of effector T- and NK-cell proliferation, increase in the CD4/CD8 T cell ratio, impairment of APC function, upregulation of PD-L1 on tumor cells and B7 family molecules on APCs, and upregulation of proangiogenic and immunosuppressive VEGF [176,177,178,179]. However, current evidence suggests that baseline AFP is not associated with objective response, but primarily functions as a prognostic biomarker, with elevated AFP, particularly if ≥400 ng/mL, being associated with an approximately 60% higher risk of mortality and 35% higher risk of disease progression [54,55,56,57,155]. More importantly, early AFP kinetics during ICI-based therapy have been associated with ORR, DCR, PFS, and OS, suggesting a potential role as an on-treatment biomarker of treatment benefit [58,59,60,61,62,63]. However, the optimal cutoffs of change in AFP and timing of assessment remain uncertain, with proposed thresholds ranging from 20% to 75%, and assessment time points from 3 to 8 weeks after treatment initiation [59,61]. A large meta-analysis suggests that a decrease in AFP ≥20% within 8 weeks may be the most informative threshold, being associated with 5-fold higher odds of achieving ORR and DCR and approximately 60% lower risks of mortality and disease progression [54]. Some studies have suggested that even a ≥10% decline in AFP may be associated with improved outcomes [54]. Conversely, an AFP increase of ≥10% in 6 weeks was associated with no disease control, with 77% sensitivity and 44% specificity, in one study [58]. Notably, in that cohort, none of the AFP non-responders who had progressive disease at the first radiological assessment subsequently achieved an objective response, suggesting that early AFP kinetics may complement radiological evaluation in identifying patients unlikely to benefit from continued treatment [58].

Composite scores incorporating both AFP and the other tumor marker des-γ-carboxyprothrombin (DCP), also known as protein-induced by vitamin K absence or antagonist II (PIVKA-II), using either baseline values or on-treatment kinetics [180,181,182,183,184] appear to have prognostic value in HCC patients treated with ICI-based regimens. However, these studies are preliminary and require prospective validation.

CRAFITY (CRP and AFP in ImmunoTherapY) is a simple prognostic score that incorporates pretreatment C-reactive protein (CRP) and AFP levels, first proposed by Scheiner et al. in 2022 [70], and subsequently evaluated in several independent cohorts [71,72,73,74,75]. Elevated CRP reflects systemic inflammatory burden and has been implicated in resistance to ICI-based therapies [185], potentially through several mechanisms, including the shift in CD4+T cell polarization toward the Th2 phenotype, which may impair anti-tumor responses [186]. The CRAFITY score is based on two variables: AFP > 100 ng/mL and CRP > 1 mg/dL, each contributing 1 point. Patients are classified into low-, intermediate-, and high-risk groups according to scores of 0, 1, and 2 points, respectively. Increasing CRAFITY scores have been associated with progressively lower ORR, PFS, and OS, and, in some studies, a higher incidence of severe adverse events [70,71,73,74]. Meta-analyses have found that a high CRAFITY score, compared with a low score, is associated with approximately a 4-fold higher risk of mortality and a 2.5-fold higher risk of disease progression [175,187]. Several modified versions of CRAFITY have subsequently been proposed, incorporating AFP and/or DCP kinetics during treatment [72,188,189], radiological features such as infiltrative tumor type, enhanced capsule, and intratumoral fat [190], or clinical features including CPT class, sarcopenia, and vascular invasion [191]. These scores show promising prognostic resolution, but require prospective validation.

Glypican-3 (GPC3) is another oncofetal protein and cell-surface heparan sulfate proteoglycan that is frequently re-expressed in HCC, where it may contribute to oncogenesis through several mechanisms, including activation of the Wnt/β-catenin and insulin-like growth factor pathways [90]. Small studies assessing GPC3 expression using immunohistochemistry (IHC) have suggested that GPC3-positive tumors, particularly those with membranous rather than cytoplasmic expression, may be associated with lower ORR and shorter OS following ICI-based therapies, suggesting a potential predictive role that warrants further validation [90]. Similarly, higher GPC3 expression, as assessed by RNA profiling, has been associated with poorer outcomes [92]. Notably, tumors with high GPC3 expression appear to have a distinct tumor microenvironment compared to tumors with low expression, characterized by a lower proportion of CD8+T cells and CD103+CD8+ tumor tissue-resident T cells, potentially providing a more immunosuppressive context for ICI-based therapy [91].

The neutrophil-to-lymphocyte ratio (NLR) and platelet-to-lymphocyte ratio (PLR) are readily available inflammatory indices that have shown potential prognostic value in ICI-based therapies. Several small retrospective studies have consistently reported that a higher pretreatment NLR is associated with shorter PFS and/or OS, although its ability to discriminate objective response has been less consistent, with most studies showing no association with ORR [64,65,66,67,68,69]. The clinical applicability of baseline NLR is, however, limited by heterogeneity of reported cutoffs, ranging from 2.4 to 5 (the most consistent cutoff being ≥5), substantial inter-individual variations; and may be influenced by comorbidities, including diabetes mellitus and hypertension, as well as concomitant medications such as antibiotics [68]. A meta-analysis of 44 studies including 5322 patients found that high NLR was associated with nearly twice the risk of mortality and a 60% higher risk of disease progression, together with approximately 50% lower odds of ORR [55]. Notably, higher NLR was also associated with an approximately 8-fold higher risk of hyperprogressive disease [55]. NLR dynamics during ICI treatment could potentially provide greater prognostic information than baseline NLR alone. An increase in NLR during treatment, particularly by more than 20% at 4 weeks or 30% at 12 weeks, has been associated with shorter PFS and OS, whereas a declining NLR has been associated with longer OS [68,192,193]. Regarding PLR, retrospective studies and meta-analyses have reported that high pretreatment PLR (with cutoffs ranging from ≥100 to ≥300, the latter being more consistent) was associated with an 80% higher risk of mortality and progression, but it was not a predictor of objective response [55,64,69,194]. Overall, NLR and PLR appear to be accessible prognostic and on-treatment markers in ICI-treated HCC, but their clinical implementation remains limited by retrospective evidence, heterogeneous cutoffs, and susceptibility to non-tumor-related factors.

Serum levels of cytokines, including IL-6 [195,196,197], IL-8 [198], IL-10 [197], and IFN-α [195], have also been investigated in patients receiving ICI-based therapies; however, the available evidence remains limited, with relatively small cohorts and heterogeneous findings, and their clinical utility has not yet been established.

Therapeutic drug monitoring, including the measurement of serum active drug concentrations and anti-drug antibodies (ADAs), is increasingly being explored to optimize treatment with monoclonal antibody-based therapies. However, evidence supporting this approach during ICI-based therapy in HCC remains limited. The incidence of ADA development varies according to the therapeutic regimen and may reach approximately one-third of patients receiving AB [199]. Small retrospective studies have suggested that high ADA titers, particularly when measured at the beginning of cycle 2, may be associated with lower ORR and shorter PFS and/or OS in patients treated with AB [76,77]. In contrast, among patients receiving DT, an analysis presented at an ASCO Annual Meeting reported that ADA development was relatively infrequent, occurring in fewer than 11% of patients, and was not associated with clinical efficacy or safety outcomes [78]. Overall, these preliminary findings suggest that ADAs may influence the pharmacokinetics and clinical activity of some ICI-based regimens; however, their role as on-treatment biomarkers for treatment optimization in HCC remains investigational.

### 3.3. Classical Biomarkers for ICI-Based Therapies

In several solid tumors, the PD-L1 expression in tumor tissue has been associated with response to ICI-based therapies [200]. However, in HCC this association appears considerably less consistent, with most pivotal clinical trials, retrospective studies, and meta-analyses failing to demonstrate a clear relationship between PD-L1 tumor expression and response or survival outcomes with ICI-based therapy [14,92,93]. Nevertheless, a meta-analysis reported 86% higher odds of ORR in patients with PD-L1-positive tumors compared with PD-L1-negative tumors, although no significant difference was observed in DCR. This association was restricted to anti-PD-1 therapy, particularly nivolumab, and to monotherapy administered in the first-line setting [201]. Consistently, an exploratory analysis of CheckMate 040, a non-comparative phase I/II study of nivolumab in HCC, reported a longer median OS among patients with PD-L1 expression ≥1% compared with those <1% (28 vs. 17 months) [202]. However, these findings should be interpreted cautiously given the exploratory nature of the analyses and the lack of consistent predictive value across studies. Interestingly, qualitative features of PD-L1 may be more relevant to ICI sensitivity than total PD-L1 abundance. Posttranslational modifications of PD-L1 can affect its stability and, consequently, the persistence of PD-L1-mediated immune suppression. Tumors with a higher abundance of the S1/CL3 glycosylated PD-L1 subtype, which is associated with greater protein stability, may remain more dependent on PD-L1 signaling for immune evasion than tumors enriched for the S2/CL3 subtype, which is more rapidly targeted for proteasome-dependent degradation. This distinction may potentially influence sensitivity to PD-1/PD-L1 blockage [203].

Serum levels of soluble PD-L1 (sPD-L1) and PD-1 (sPD-1) have been investigated as circulating surrogates for the PD-1/PD-L1 pathway. In HCC, sPD-L1 levels have been shown to correlate with PD-L1 expression on tumor cells and are associated with overall survival [204,205]. However, evidence specifically evaluating soluble PD-1/PD-L1 as biomarkers for ICI-based therapy remains limited. In a small cohort of patients with HCC receiving nivolumab, sPD-1 and sPD-L1 levels changed during treatment; however, these changes were not significantly associated with clinical outcomes [206].

MSI-H/dMMR is an established predictor of responsiveness to ICI across several solid tumors [207,208]. However, it is exceedingly uncommon in HCC, occurring in only 0.2–2.4% of patients [209,210,211]. Consequently, studies evaluating its predictive value in ICI-based therapy for HCC are limited by the very small number of biomarker-positive patients and are underpowered to establish associations with treatment outcomes. Similarly, a high tumor mutational burden (TMB-H) is rare in HCC, being reported in less than 1% of patients [209]. A meta-analysis suggested that TMB-H may be associated with longer OS among patients receiving ICI-based therapies [94]. Overall, although MSI-H/dMMR and TMB-H status are biologically compelling biomarkers of ICI sensitivity, their very low prevalence and limited validation substantially restrict their clinical applicability in HCC.

### 3.4. Molecular Classification and Mutational Profiling

Montironi et al. [100] proposed a molecular classification of HCC into two major classes: inflamed and non-inflamed classes. The inflamed class accounted for 37% of patients and was characterized by increased IFN-γ and PD-1 signaling, together with overexpression of lymphocyte chemokines such as CXCL9 and CXCL10. This class was further subdivided into immune-active, immune-exhausted, and immune-like (characterized by CTNNB1, encoding for β-catenin, mutations) subclasses. The non-inflamed class comprised the intermediate subclass (43% of the patients, characterized by TP53 mutations and chromosomal losses), and the immune-excluded subclass (20%, characterized by CTNNB1 mutations and PTK2 overexpression). Activating CTNNB1 mutations, which lead to overactive β-catenin signaling, have been implicated in immune exclusion through defective recruitment of APCs and impaired T cell function, providing a potential mechanism of resistance to ICIs [212]. Notably, CTNNB1 mutations are not restricted to the immune-excluded subclass: approximately two-thirds of CTNNB1-mutated tumors belonged to the immune-excluded subclass, whereas the remaining third occurred in the immune-like subclass. This observation suggests that the biological consequences of CTNNB1 activation may depend on the broader immune context of the tumor. The authors also developed a 20-gene signature that captured approximately 90% of inflamed tumors. In an external cohort of ICI-treated patients, responders exhibited higher expression of the inflamed signature than non-responders, suggesting that the inflamed phenotype was enriched for ICI-responsive tumors. Although CTNNB1-driven β-catenin activation is mechanistically associated with immune exclusion and ICI resistance, in a multicenter cohort of 111 HCC patients treated with ICI-based therapies, CTNNB1 mutational status alone was not sufficient to predict treatment resistance [213]. Rather, the impact of CTNNB1 mutations appeared to depend on the underlying immune phenotype: CTNNB1-mutated tumors within the immune-excluded phenotype were more likely to be resistant to ICI-based therapy, whereas CTNNB1 mutations occurring within the immune-like phenotype did not confer the same degree of resistance, potentially because the pre-existing inflamed immune microenvironment outweighed the immunosuppressive effect of β-catenin activation [213]. Consistent with these findings, a large study of 1306 patients with HCC reported that higher β-catenin RNA expression was associated with a shorter duration of ICI-based treatment [214]. β-catenin pathway activity can also be assessed by IHC, using β-catenin or glutamine synthetase as surrogate markers. A pilot study suggested that patients with low β-catenin expression and high intratumoral CD8+ T cell density may derive greater benefit from DT. However, IHC assessment of the β-catenin pathway activation has limited ability to identify underlying CTNNB1 mutations. Even glutamine synthetase IHC, which performed better than β-catenin IHC, showed only approximately 78% sensitivity and 83% specificity for CTNNB1 mutations [215], limiting its potential use as a surrogate for CTNNB1 mutational status in clinical practice.

A different molecular classification was proposed by Wang et al., who subdivided HCCs into three phenotypes: cholangiocyte-like, hepatocyte-like, and progenitor-like, which showed progressively lower ORR and progressively less benefit in PFS and OS with AB compared to sorafenib, in the GO30140 and IMbrave150 trials [101]. The cholangiocyte-like phenotype was characterized by a strong immune infiltrate, absence of hepatocellular differentiation markers Hep/Par and ARG1, and positivity for fibroblast activating protein (FAP). Conversely, the hepatocyte-like phenotype was positive for Hep/Par and ARG1 and cytochrome P450 enzymes, and had an intermediate degree of immune infiltrate. Finally, the progenitor-like phenotype was characterized by an immune-desert microenvironment and expression of GPC3. These findings suggest that both the tumor differentiation state and immune context may influence the efficacy of AB. Therefore, it would be of interest to investigate whether these molecular phenotypes could be approximated using an IHC-based panel comprising HepPar1, ARG1, FAP, and GPC3, and to validate its prognostic and predictive value in large independent cohorts of patients with HCC receiving ICI-based therapies.

### 3.5. Molecular Signatures from Bulk, Single Cell, and Spatial Transcriptomics

One of the most comprehensive analyses of the molecular correlates of clinical response to AB was conducted by Zhu et al. [92]. The authors performed an integrated molecular analysis of tumors from 358 patients enrolled in the GO30140 and IMbrave150 trials, in which patients received AB, atezolizumab monotherapy, or sorafenib. Tumors responding to AB were characterized by high CD274 expression (encoding PD-L1), increased intratumoral CD8+ T cell density, and a T-effector (Teff) signature, consisting of the presence of pre-existing antitumor immunity. Conversely, a high Treg-to-Teff ratio and high expression of oncofetal genes GPC3 and AFP were associated with resistance to AB. Furthermore, high expression of VEGF receptor 2 (KDR) together with Treg and myeloid inflammation signatures was associated with greater benefit from the addition of bevacizumab to atezolizumab, supporting the concept that bevacizumab contributes not only through inhibition of VEGF-mediated angiogenesis but also through modulation of the immunosuppressive tumor microenvironment. Importantly, the authors developed a transcriptomic AB response signature (ABRS) comprising 10 genes (CXCR2P1, ICOS, TIMD4, CTLA4, PAX5, KLRC3, FCRL3, AIM2, GBP5, and CCL4). ABRS captured a broader immune-active transcriptional phenotype involving T cell activation and innate immunity pathways, and was associated with clinical benefit. Patients with high ABRS had longer PFS and OS, with HR approximately 0.49 and 0.26, respectively.

A multicenter cohort of 122 patients treated with AB was used to develop an artificial intelligence (AI)-based digital pathology model, dubbed ABRS-P, which estimates the transcriptomic ABRS directly from histological slides [102]. The authors found a good correlation between ABRS-P and transcriptomic ABRS, although this correlation was weaker in younger patients (≤60 years) and those with cirrhosis. Interestingly, higher ABRS-P was associated with PFS, but not with OS. ABRS-P represents a proof of concept for a rapid, slide-based surrogate of a transcriptomic biomarker, potentially enabling molecular information associated with ICI response to be obtained from routinely available histological material without requiring RNA sequencing. However, its reduced performance in younger and cirrhotic patients and its lack of association with OS highlight the need for further validation before clinical implementation.

A different transcriptomic signature of response to AB was subsequently derived from a single-cell transcriptomic study by Lim et al. [103]. Using single-cell RNA sequencing (scRNA-seq), the authors identified distinct cellular and transcriptional features between responders and non-responders to AB. Responders exhibited T- and NK-cell populations with higher cytotoxic and exhaustion programs, enrichment of proinflammatory TAMs, increased CD1c+ dendritic cells, and endothelial cells with enhanced antigen-presenting functions. In contrast, non-responders showed enrichment of resting TAMs and endothelial cells characterized by increased cell-projections and angiogenic programs. The authors subsequently integrated the scRNA-seq data with bulk RNA-sequencing data to develop a 10-gene (GZMH, PRF1, CTLA4, PDCD1, ICOS, LAG3, CCL4, CD1C, FCER1A, and CD1E) AB response signature, termed sc_ABRS. Higher sc_ABRS were associated with longer PFS, but not OS. In this cohort, the predictive performance of sc_ABRS was comparable to that of the original ABRS [92].

A recent study by Cappuyns et al. further sheds light on the mechanisms underlying AB responsiveness, suggesting that response is not necessarily driven by a single biological process but may occur through distinct molecular routes [216]. The authors analyzed 422 patients, of whom 317 were treated with AB, 47 with atezolizumab monotherapy, and 58 with sorafenib. Using scRNA-seq, the authors derived gene signatures representing 21 cell phenotypes within the HCC tumor microenvironment, which were subsequently applied to bulk transcriptomic data. Patients were stratified according to the presence of three immune cell-associated signatures: exhausted CD8+Tex cells, terminally differentiated effector memory CD8+Temra cells, and CXCL10+TAMs, thereby defining immune-positive and immune-negative tumors. Among immune-positive tumors, AB-responsive tumors were characterized by a low abundance of CD14+ monocytes and TREM2+ TAMs, whereas immune-positive non-responders were enriched for these immunosuppressive myeloid populations. This immune-positive/responsive phenotype accounted for approximately 40% of responders, whereas the immune-positive/resistant phenotype accounted for approximately 25% of non-responders. Among immune-negative tumors, a second route to response was identified: tumors with low expression of NRP1, encoding neuropilin-1, were classified as an angiogenesis-driven phenotype and represented approximately 60% of responders. In contrast, immune-negative non-responders were characterized by high NRP1 expression and increased Notch pathway activity, accounting for approximately 75% of non-responders. Overall, the authors used scRNA-seq-derived cell-type signatures together with NRP1 expression and Notch pathway activity to molecularly stratify tumors into distinct response phenotypes, including an immune-competent phenotype, an angiogenesis-driven phenotype, and a resistant phenotype.

Another study investigated whether the oncofetal reprogramming of the HCC tumor microenvironment could be linked to response to AB [217]. Li et al. identified three major oncofetal populations: PLVAP+ endothelial cells, FOLR2+ TAMs, and POSTN+ CAFs. Using spatial transcriptomic and cell–cell communication analyses, the authors demonstrated that these populations co-localized and interacted within the tumor, forming an onco-fetal neighborhood. Subsequently, the authors developed an onco-fetal score representing the cellular abundance of these three populations, estimated from bulk RNA-sequencing data. They then investigated the relationship between this cellular ecosystem and the molecular phenotype associated with AB response using the AB response signature (ABRS) described by Zhu et al. [92]. Interestingly, a higher onco-fetal score was associated with a higher ABRS, suggesting that tumors enriched in the onco-fetal ecosystem also exhibit molecular features associated with response to AB.

A multicenter cohort from Europe and the US included 111 HCC patients treated with ICI-based therapies, of whom 83 had adequate pretreatment tissue for transcriptomic analysis [213]. Among these 83 patients, 28 received ICI-based therapy in the first-line setting, whereas 55 received it in the second- or third-line setting following sorafenib. In patients treated in the first-line setting, responders exhibited upregulation of IFN-γ signaling and major histocompatibility complex (MHC)-II-related antigen presentation pathways. The authors developed an 11-gene signature, dubbed IFNAP, comprising genes involved in IFN-γ signaling (STAT1 and GBP1), antigen presentation (B2M, HLA-DRB5, HLA-DRA, HLA-DPA1, HLA-DPB1, HLA-DQA1, HLA-DQB1, and HLA-DMA), and chemotaxis (CXCL9). High IFNAP scores were associated with treatment response and improved survival specifically in patients treated with ICI-based therapies in the first-line setting, but not in those previously exposed to sorafenib. This highlights the need to repeat liver when evaluating biomarkers in later treatment lines. Interestingly, IFNAP was not primarily associated with the overall extent of immune infiltration, but rather with its cellular composition. High IFNAP scores were associated with enrichment of plasma cells, activated CD4+ memory T cells, and M1-like TAMs, whereas low IFNAP scores were associated with enrichment of immunosuppressive Tregs.

Notably, patients with multiple nodules may show molecular internodular variability, and consequently, ICI responsiveness variability. Huang et al. [218] analyzed 45 HCC nodules from 12 patients and found that, in patients with multifocal HCC, smaller nodules were more sensitive to ICI-based therapies than larger nodules. Smaller nodules exhibited greater immune-cell infiltration (including CD8+ T cells and M1-like TAMs) and upregulation of an IFN-γ signature predictive of response to anti-PD1 therapy [219].

Beyond immune cell composition, the spatial organization and localization of specific immune populations within the tumor also appear to influence ICI responsiveness. In a cohort of 94 patients treated with AB, tumor biopsies were evaluated using IHC, bulk RNA-seq, flow cytometry, and multiplexed imaging. Although CD8+T cell density was not associated with treatment outcomes, the spatial distribution of PD-L1+CD8+T cells was informative. Among patients with a high proportion of PD-L1+ CD8+ cells (i.e., ≥58%), a diffuse distribution of these cells throughout the tumor parenchyma was associated with longer PFS, whereas a more localized distribution within the parenchyma or predominant localization within the fibrous stroma (hence spatially separated from tumor cells) was associated with shorter PFS [95]. Similar associations between the spatial distribution of intratumoral CD8+T cells and ICI responses have been reported by other groups [96]. Other studies have evaluated the density of intratumoral CD8+T cells by IHC. High CD8+ tumor-infiltrating lymphocyte (TIL) density, defined as >15.9 cells per high-power field, was associated with higher ORR and longer PFS and OS following treatment with AB or DT [97,98]. Conversely, another small study found that CD8+T cell density alone was not discriminative, whereas spatial proximity between CD8+T cells and PD-L1+TAMs was informative: colocalization of CD8+T cells within 25 μm of PD-L1+TAMs was associated with objective response and longer PFS in patients treated with AB, but not in those treated with lenvatinib [99]. Macrophage organization may similarly contribute to immune exclusion. Macrophage-coated tumor clusters (MCTCs) are aggregates in which TAMs surround tumor-cell clusters, creating a physical barrier that may sequester CD8+T cells outside the tumor-cell compartment and thereby limit effective tumor-cell engagement. The presence of MCTCs was associated with lower ORR following ICI-based therapy, supporting a potential role for this spatial architecture in ICI resistance [220]. MCTCs were enriched in TREM2+TAMs [220], a macrophage population that has also been associated with non-response to ICI-based therapies in other studies [221]. Conversely, tertiary lymphoid structures (TLS), which can be histologically identified as organized aggregates containing B cells, T cells, and dendritic cells resembling secondary lymphoid organs, represent a spatially organized immune microenvironment. TLS has been associated with the inflamed HCC subtype [222] and appears to be associated with improved outcomes following ICI-based therapies [223,224].

### 3.6. Liquid Biopsy

Several experimental approaches are currently being investigated to identify minimally invasive biomarkers of response to ICI-based therapies, including immune cell profiling of peripheral blood mononuclear cells (PBMCs), analysis of extracellular vesicles, circulating tumor DNA (ctDNA), and circulating tumor cells (CTCs).

To date, no robust PBMC-based predictive biomarker has been established for ICI-based therapies in HCC. The available evidence is largely derived from small and heterogeneous studies, which differ in treatment regimens, sampling time points, and immune cell populations analyzed. Nevertheless, PBMCs are readily accessible and can be repeatedly collected, making them particularly attractive for serial longitudinal monitoring of treatment-induced changes in the systemic immune compartment. Some studies have reported associations between baseline or on-treatment PBMC characteristics and clinical outcomes [225,226,227,228,229,230,231,232]. For example, a higher baseline proportion of PD-L1+CD4+T cells was associated with response to tremelimumab [104]; increased baseline senescent CD28−CD57+CD8+T cells was associated with non-response to AB [105]; lower abundance of PD-L1+B cells at baseline or PD-L1+monocytes following nivolumab was associated with stable disease [106]; and lower abundance of NK-like innate lymphoid cells (ILCs) expressing the cytotoxicity marker NKp80/KLRF1 was associated with shorter PFS following DT [107].

A study using an unbiased high-throughput plasma proteomic screen in 78 patients treated with AB reported that plasma carbonic anhydrase 9 (CA9) concentrations >188.7 pg/mL were independently associated with lower ORR and shorter PFS and OS [233]. These findings have biological plausibility, as CA9 can be expressed by tumor cells, and tumor CA9 expression has been reported to correlate with circulating plasma levels. CA9 is an important regulator of tumor-cell pH and contributes to extracellular acidification while facilitating the maintenance of a relatively alkaline intracellular pH. This altered pH homeostasis can promote tumor-cell survival and progression and may contribute to an immunosuppressive tumor microenvironment, potentially reducing the effectiveness of ICI-based therapies. However, the discriminatory performance of plasma CA9 was limited, as a substantial proportion of patients with high CA9 concentrations still achieved an objective response.

Tumor-derived extracellular vesicles (EVs) facilitate intercellular communication and may contribute to immune evasion and modulation of ICI responsiveness. Egerer et al. reported that HCC patients who responded to AB had smaller EVs at baseline and exhibited a decline in EV size during treatment [108]. In a larger study of 202 patients with HCC, Gorgulho et al. found an enrichment of immune checkpoint molecules, including PD-1, PD-L1, and CTLA-4, in the EV fraction compared to the EV-depleted plasma fraction [109]. Lower baseline levels of EV-associated immune checkpoints and a subsequent decline during treatment were associated with better clinical outcomes in patients receiving ICI-based therapies but not in those receiving TKI therapy [109]. These findings suggest that EV-associated immune checkpoint molecules may reflect a systemic mechanism of immune regulation that is particularly relevant to the ICI response.

Techniques to evaluate ctDNA and CTCs have also been investigated in this context, although the available evidence remains preliminary. ctDNA consists of tumor-derived DNA fragments released into the circulation, primarily through tumor-cell death, and represents only a small fraction of total cell-free DNA (cfDNA). ctDNA can be distinguished from non-tumor-derived cfDNA through the detection of cancer-specific molecular alterations, including mutations, copy number alterations, and epigenetic modifications such as aberrant CpG methylation [234]. Higher levels of cfDNA and ctDNA have been associated with greater tumor burden and, inconsistently, with lower ORR and shorter OS in patients receiving ICI-based therapy [110,111,112,113,114]. However, most studies have failed to demonstrate consistent associations between specific mutations detected in ctDNA, including in the TERT promoter, TP53, CTNNB1, and other genes in the Wnt pathway, and ICI responsiveness [110,111,114,235]. In contrast, a small study in a Japanese population [111] reported a 3-fold higher risk of death in patients harboring mutations in the TERT promoter. This association may partly reflect underlying advanced liver disease, as TERT promoter mutations are associated with cirrhosis [110] and can be detected even in premalignant dysplastic or regenerative nodules in cirrhotic livers [236]. Furthermore, maximum variant allele frequency (VAF) has been inconsistently [235,237] associated with shorter PFS and OS in HCC patients treated with AB but not in those treated with TKI [238]. Conversely, an early decline in VAF following treatment initiation was associated with higher ORR and longer PFS [237]. Similarly, ctDNA mutation burden increased with more advanced tumor stage and its persistence following AB treatment was associated with a higher probability of radiological progression [114].

CTCs, which are shed from primary or metastatic tumors into the bloodstream, are extremely rare cells that can be detected in the blood. Their abundance has been associated with tumor burden, tumor invasiveness, and metastatic potential [234]. Small studies have reported that, among HCC patients receiving ICI-based therapy, a higher proportion of PD-L1+CTCs, as well as a rapid decline in PD-L1+CTCs following treatment initiation, was associated with a greater likelihood of achieving ORR and with longer PFS or OS [115,116,117].

### 3.7. Microbiota

The gut-liver axis is characterized by bidirectional interactions between the gut microbiota and the liver, facilitated by their anatomical proximity and direct connection through portal circulation. Dysbiosis, broadly referring to alterations in the composition and function of the gut microbiota, together with disruption of intestinal permeability, is an established contributor to hepatocarcinogenesis [239] and a modulator of hepatic immune function [240]. An increasing, although not yet validated, body of evidence suggests that the gut microbiota may also modulate outcomes in patients with HCC receiving ICI-based therapies.

Gut microbiota can be perturbed by several commonly used medications, including antibiotics and proton pump inhibitors (PPIs). Several cohorts have evaluated the association between previous antibiotic exposure and outcomes following ICI-based therapies [169,241,242,243,244,245]. A recent meta-analysis of eight studies reported that antibiotic exposure was associated with an approximately 25% higher risk of disease progression and a 50% higher risk of mortality, although there was no association with ORR [246]. Interestingly, in contrast to the findings reported in other solid tumors, the association between antibiotic exposure and outcomes appeared to be similar in HCC patients, regardless of the treatment modality, including ICI-based therapy, TKIs, or placebo [243]. This might reflect an association with disease severity or overall prognosis rather than a treatment-specific effect predictive of ICI responsiveness. Regarding PPI exposure, different cohorts and meta-analyses have not demonstrated a consistent association with outcomes following ICI-based therapies in HCC [244,247,248].

Studies evaluating the composition of the gut microbiota in relation to ICI response remain exploratory and are characterized by high heterogeneity in methodology and patient populations [38]. Nevertheless, poorer outcomes have been associated with features of gut dysbiosis, including reduced microbial diversity [118,119,249], skewed Firmicutes/Bacteroidetes and *Prevotella*/*Bacteroides* ratios; reduced abundance of potentially beneficial bacteria such as members of the genus *Ruminococcus*, *Akkermansia muciniphila*, and *Faecalibacterium prausnitzii*; and enrichment of potentially pathogenic bacteria, including some members of the genus *Bacteroides* and the Enterobacteriaceae family [118,120,121,122]. Certain potentially pathogenic or proinflammatory bacteria, including *Bacteroides* and *Escherichia coli*, have been associated with impaired anti-tumor immune responses, including reduced lymphocyte infiltration and impaired antigen presentation [118]. Conversely, *Akkermansia muciniphila* has been shown in preclinical models to elicit systemic immune responses and remodel the tumor microenvironment, thereby potentiating ICI-responsiveness [123]. Microbiota dynamics with an early shift to more favorable microbial profiles following ICI-based treatment initiation were also associated with improved responses [124].

Pretreatment lower levels of fecal calprotectin, a marker of intestinal inflammation, were also associated with response to ICI-based therapies [121], suggesting that a less inflammatory intestinal environment may be associated with greater treatment benefits.

Behrens et al. used phage immunoprecipitation sequencing technology to measure viral and bacterial antibodies in 45 HCC patients receiving ICI-based therapies and developed a prognostic score comprising 23 reactive microbial peptides. A high-risk score was associated with approximately a 4-fold higher risk of disease progression and a greater than 80-fold higher risk of mortality [125]. Interestingly, in this cohort, Epstein–Barr virus (EBV) appeared to negatively impact survival [125].

### 3.8. Imaging and Radiomics

Radiomics takes advantage of advanced computational technologies, frequently incorporating AI or machine learning approaches, to extract quantitative information from medical images (including CT, MRI, and less commonly, ultrasound) that would not be visible to the naked eye, including tumor shape, texture, intensity, and wavelet-transformed characteristics, thereby providing quantitative measures of intratumoral heterogeneity. Different groups have investigated radiomic approaches to predict outcomes following ICI-based therapies in HCC, with promising results [250,251,252,253,254,255,256,257].

Studies using MRI with the hepatobiliary-specific contrast agent gadolinium-ethoxybenzyl-diethylenetriamine pentaacetic acid (Gd-EOB-DTPA; Primovist^®^ or Eovist^®^) have investigated the hepatobiliary relative enhancement ratio (RER), calculated from the signal intensity of intrahepatic HCC nodules relative to the background liver. A higher RER has generally been associated with an immune-excluded tumor phenotype and, in several studies, with a lower likelihood of response to ICI-based therapies and poorer survival [80,81], although results have been inconsistent, particularly among patients treated with AB [82,83,84]. The biological rationale is that HCCs harboring activating CTNNB1 alterations and increased Wnt/β-catenin signaling can overexpress the organic anion-transporting polypeptide 1B3 (OATP1B3), resulting in increased uptake of hepatobiliary-specific contrast agents and greater hepatobiliary-phase enhancement [85]. A recent meta-analysis of five studies including 253 patients reported that RER ≥ 0.9 was associated with an approximately 6-fold higher risk of mortality following ICI-based therapies [86]. However, this association was not statistically significant among patients receiving regimens combining ICIs with anti-VEGF therapy, raising the possibility that anti-VEGF treatment may partially overcome the immune-excluded phenotype associated with high RER [86].

18F-fluorodeoxyglucose (FDG) PET-CT is not routinely recommended for HCC staging because FDG uptake has limited sensitivity and is observed in only approximately 40% of HCCs in some studies [258]. Nevertheless, FDG-PET is gaining interest as a potential marker of tumor biology, as FDG uptake appears to be associated with more aggressive features, including poor histological differentiation, increased proliferative activity, stem cell-like characteristics, and epithelial–mesenchymal transition (EMT) features [259]. Furthermore, FDG-PET-positive HCCs appear to be inflamed but immune-exhausted in phenotype [260], and have been associated, in small studies, with worse response to AB, with lower ORR and lower PFS, albeit with better response to dual CTLA-4/PD-L1 DT treatment [87,88,89].

## 4. Conclusions

The development of robust and clinically validated predictive biomarkers for response to ICI-based therapies remains an unmet need in HCC. Nevertheless, vibrant research activity is ongoing, which may provide clinically useful tools in the near future. Importantly, the quest for biomarkers has already generated substantial knowledge regarding the biological mechanisms underlying ICI resistance and has provided hypotheses for the development of strategies aimed at overcoming these mechanisms.

In the meantime, several readily available clinical prognostic tools can contribute to the management of patients with HCC, including measures of liver function and simple inflammatory markers, such as the NLR. Furthermore, the kinetics of tumor markers, such as AFP, may provide an early indication of treatment efficacy, potentially allowing timely reassessment of treatment strategy in patients who are unlikely to benefit.

Multiomics studies have provided important insights that may pave the way for the development of simple clinically applicable histological scores. Such scores could potentially integrate complementary features of the tumor microenvironment, including the abundance and spatial localization of CD8+T cells, together with markers of lineage differentiation skewed to cholangiocyte, hepatocyte, or progenitor-like phenotypes.

Imaging approaches may also evolve into tools for treatment selection. For example, hepatobiliary-specific contrast-enhanced MRI could potentially help identify patients with imaging features of Wnt/β-catenin-associated immune exclusion who may benefit more from the addition of anti-VEGF therapy to ICI. Conversely, FDG-PET may potentially help identify distinct tumor phenotypes that could benefit from alternative treatment strategies, including dual ICI therapy or TKI-based approaches rather than AB. However, these hypotheses require prospective validation before imaging biomarkers can be incorporated into treatment selection.

In conclusion, although the “million-dollar question” of how to reliably predict the response to ICI-based therapies in HCC remains unanswered, the rapidly expanding biomarker field is progressively transforming our understanding of the determinants of ICI sensitivity and resistance. The integration of clinical, pathological, molecular, spatial, circulating, microbiome, and imaging biomarkers may ultimately enable a more precise selection of treatment for individual patients.

## Figures and Tables

**Table 1:** Summary of the main studies for ICI regimens approved for first-line HCC treatment.

	IMbrave 150 [5,6]	HIMALAYA [7,8]	CheckMate 9DW [9]	CARES-310 [10]	ORIENT-32 [11]
Treatment Arms	Atezolizumab + Bevacizumab vs. Sorafenib	Durvalumab + Tremelimumab (STRIDE) vs. Sorafenib	Nivolumab + Ipilimumab vs. Sorafenib or Lenvatinib	Camrelizumab + Rivoceranib vs. Sorafenib	Sintilimab + IBI305 (Bevacizumab biosimilar)
N	501	782	668	543	571
ORR (%)	30.0 vs. 11.0	20.1 vs. 5.1	36 vs. 13	25.4 vs. 5.9	21.0 vs. 4.0
CR (%)	8.0 vs. <1	3.1 vs. 0	7 vs. 2	1.1 vs. 0	1.0 vs. 0
DCR (%)	74 vs. 55	60.1 vs. 60.7	68 vs. 75	78.3 vs. 53.9	72.0 vs. 64
Median PFS (months)	6.9 vs. 4.3	3.8 vs. 4.1	9.1 vs. 9.2	5.6 vs. 3.7	4.6 vs. 2.8
Median OS (months)	19.2 vs. 13.4	16.4 vs. 13.8	23.7 vs. 20.6	23.8 vs. 15.2	NR vs. 10.4
Any grade TRAE (%)	86 vs. 95	75.8 vs. 84.8	84 vs. 91	88 vs. 68	88.7 vs. 93.5
Grade 3–4 TRAE (%)	43 vs. 46	25.8 vs. 36.9	41 vs. 42	25 vs. 7	33.7 vs. 35.7

N, number of patients enrolled; ORR, objective response rate; CR, complete response; DCR, disease control rate; PFS, progression-free survival; OS, overall survival; TRAE, treatment-related adverse event.

**Table 2:** Biomarkers and tools to assess/predict response to ICI-based therapies in HCC.

Biomarker	Classification	Treatment Regimen	Evidence	Clinical Application
Clinical and laboratorial parameters				
CPT score and ALBI	Prognostic	ICI-based therapies in general, but stronger evidence for AB	Real-world retrospective studies/post hoc trials [39,40,41,42,43,44,45,46,47,48,49,50,51,52,53]	Tools to assess liver function. Allow survival stratification and help identify patients whose poor prognosis or clinical condition may preclude treatment.
AFP	Baseline: Prognostic Kinetics: On treatment	ICI-based therapies in general, but stronger evidence for AB	Retrospective studies/post hoc trials [54,55,56,57,58,59,60,61,62,63]	AFP ≥ 400 ng/mL is associated with higher mortality. Early AFP kinetics and response assessment: ↓ ≥20% at 8 weeks is associated with ↓ 60% mortality risk; ↑ ≥10% at 6 weeks is associated with no disease control (Se 77%, Sp 44%). Kinetics may support closer surveillance and earlier reassessment. Needs prospective studies and harmonization of cutoffs and timing.
NLR	Baseline: Prognostic Kinetics: On treatment	ICI-based therapies in general, but stronger evidence for AB	Multiple independent retrospective and even prospective studies [64,65,66,67,68,69]	Readily available inflammatory index associated with OS. Heterogeneity in baseline cutoffs, the most consistent being ≥5. Early NLR kinetics and response assessment: an ↑ in NLR at weeks 4–6 is associated with worse survival and may support closer surveillance and earlier reassessment. Needs prospective studies and harmonization (cutoffs, timing).
CRAFITY score	Prognostic	ICI-based therapies in general, but stronger evidence for AB	Multiple independent retrospective studies [70,71,72,73,74,75]	Easy tool, ready to use, with prognostic information. A high CRAFITY score was associated with 4-fold ↑ risk of mortality and 2.5-fold ↑ risk of disease progression.
Anti-drug antibodies (ADAs)	Potentially On treatment?	AB, less evidence for DT	Small retrospective studies [76,77,78]	High titers of ADA at cycle 2 of AB may be associated with lower ORR and OS, while those associations were not found for DT. Their role in treatment optimization is still to be determined.
Imaging				
RECIST 1.1 mRECIST 1.1	On treatment	Bulk of the studies performed in patients treated with TKI	Prospective studies with external validation [79]	Standard evaluation of response. Small retrospective studies found that mRECIST 1.1 detected more responses, but mRECIST 1.1 and RECIST 1.1 presented similar prognostic discriminative capacity.
MRI radiomics	Potentially predictive?	ICI-based therapies in general, but stronger evidence for AB	Small retrospective cohorts [80,81,82,83,84,85,86]	Exploratory/hypothesis generating. RER may differentially associate with outcomes according to treatment regimen, with RER ≥ 0.9 being associated with ↑ mortality following ICIs, but not when ICIs are combined with anti-VEGF treatment, although results for AB have been inconsistent. Prospective validation is required before clinical application for treatment selection.
FDG-PET CT	Potentially predictive?	AB and DT	Small retrospective studies [87,88,89]	FDG-PET+ HCC have been associated with poorer response to AB and better response to dual ICIs. Prospective validation is required before clinical application for treatment selection.
Histology				
GPC3 IHC	Prognostic Potentially predictive?	AB	Single-center retrospective cohorts [90,91]; lack of external validation.	While promising, evidence remains insufficient to establish the value of GPC3 as a predictive biomarker for response to ICI-based therapies. Lack of standardized IHC scoring cutoff. Needs prospective validation and comparison between ICI- and TKI-based therapies.
PD-L1 IHC	No established role as a predictive biomarker	ICI-based therapies in general, but stronger evidence for AB	Retrospective studies, post hoc trials and meta-analyses [14,92,93]	A meta-analysis suggested PD-L1+ tumors might be associated with ↑ ORR (but not OS), although only for anti-PD-1 monotherapy. Limited by low PD-L1 expression in HCC (<20% of cases) and heterogeneous distribution across the tumor.
MSI-H/dMMR and high TMB	No established role as a predictive biomarker	ICI-based therapies in general, but stronger evidence for AB	Retrospective studies [94]	MSI-H/dMMR is present in only 0.2–2.4% and high TMB in <1% of HCCs. Limited by very low prevalence and lack of validation.
IHC for CD8+T-cells	Potentially predictive?	AB and DT	Retrospective studies [95,96,97,98,99]	Exploratory/hypothesis generating. High density of CD8+T-cells, particularly if colocalizing with PD-L1+TAMs, and diffuse distribution of PD-L1+CD8+T-cells were associated with ↑ ORR and ↑ OS.
Molecular classification and Molecular signatures				
Inflamed vs. non-inflamed	Potentially predictive?	AB	Retrospective study [100]	Exploratory/hypothesis generating. Higher expression of inflamed signature in responders to ICIs.
Differentiation: cholangiocyte-, hepatocyte-, progenitor-like	Potentially predictive?	AB	Retrospective study [101]	Exploratory/hypothesis generating. Cholangiocyte-, hepatocyte-, and progenitor-like, associated with progressively ↓ ORR and ↓ OS benefit with AB vs. sorafenib. In the future, it may pave the way for an IHC-based panel comprising HepPar1, ARG1, FAP, and GPC3.
Molecular signatures, for example ABRS	Potentially predictive?	AB	Retrospective studies [92,102,103]	Exploratory/hypothesis generating. Transcriptomic signature of 10 genes, for prediction of response to AB—high score was associated with ↓ 50% risk of progression and ↓ 75% risk of mortality [92].
Liquid biopsy				
PBMC-based	Potentially predictive? On-treatment?	ICI-based therapies in general, but stronger evidence for AB and N	Retrospective studies [104,105,106,107]	Exploratory/hypothesis generating. No robust PBMC-based biomarker has yet been identified.
Tumor-derived EV	Potentially predictive? On-treatment?	ICI-based treatments in general, but stronger evidence for AB	Retrospective studies [108,109]	Exploratory/hypothesis generating. Reports that smaller EV and lower levels of EV-associated immune checkpoints might be associated with better outcomes.
ctDNA	Baseline: Prognostic Kinetics: On-treatment?	ICI-based treatments in general, but stronger evidence for AB	Retrospective studies [110,111,112,113,114]	Exploratory/hypothesis generating. Dynamic ctDNA clearance showed the strongest signal: higher clearance was associated with higher odds of response.
CTC	Potentially predictive? On-treatment?	ICI-based treatments in general	Small retrospective studies [115,116,117]	Exploratory/hypothesis generating. Higher PD-L1+CTCs and their rapid decline following treatment initiation were associated with a ↑ ORR, PFS, and OS.
Microbiota				
Gut microbiota bacteria profiling	Response-correlative	ICI-based treatments in general	Small retrospective and prospective cohorts [118,119,120,121,122,123,124]	Exploratory/hypothesis generating. High heterogeneity of studies. High abundance of *Akkermansia muciniphila*, Ruminococcaceae and *Faecalibacterium*, and low abundance of Proteobacteria and Enterobacteriaceae have been associated with ↑ response.
Anti-viral/bacterial antibody score	Prognostic?	ICI-based treatments in general	Prospective study [125]	Exploratory/hypothesis generating. High score comprising 23 reactive microbial peptides was associated with ↑ disease progression and ↓ OS.

AB, atezolizumab plus bevacizumab; ABRS, atezolizumab plus bevacizumab response score; ADA, anti-drug antibodies; AFP, α-fetoprotein; CPT, Child Pugh Turcotte; CTC, circulating tumor cells; ctDNA, circulating tumor DNA; DT, durvalumab plus tremelimumab; EV, extracellular vesicles; GPC3, glypican-3; ICI, immune-checkpoint inhibitors; IHC, immunohistochemistry; MSI-H/dMMR, microsatellite instability-high/mismatch repair deficiency; N, nivolumab; NLR, neutrophils-to-lymphocytes ratio; ORR, objective response rate; OS, overall survival; PBMC, peripheral blood mononuclear cells; PD-L1, programmed death ligand-1; PFS, progression free survival; RER, relative enhancement ratio; Se, sensitivity; Sp, specificity; TMB, tumor mutational burden; ↓, decrease, ↑, increase.

## Data Availability

No new data were created or analyzed in this study. Data sharing is not applicable to this article.

## Abbreviations

The following abbreviations are used in this manuscript: AB, atezolizumab plus bevacizumab; ABRS, AB response signature; ADA, anti-drug antibodies; AFP, α-fetoprotein; AI, artificial intelligence; ALBI, albumin-bilirubin; APC, antigen-presenting cells; BMI, body mass index; CA9, carbonic anhydrase 9; CAFs, cancer-associated fibroblasts; cfDNA, cell-free DNA; CTC, circulating tumor cells; ctDNA, circulating tumor DNA; CRAFITY; CRP & AFP in ImmunoTherapY; CRP, C-reactive protein; CTLA-4, cytotoxic T-lymphocyte-associated protein-4; DCP, des-γ-carboxyprothrombin; DCR, disease control rate; DT, durvalumab plus tremelimumab; EMT, epithelial–mesenchymal transition; EV, extracellular vesicles; GPC3, glypican-3; FDG, fluorodeoxyglucose; Gd-EOB-DTPA, gadolinium-ethoxybenzyl-diethylenetriamine pentaacetic acid; HCC, hepatocellular carcinoma; ICI, immune-checkpoint inhibitors; IHC, immunohistochemistry; irAE, mmune-related adverse events; MASLD, metabolic dysfunction-associated steatotic liver disease; MCTCs, macrophage-coated tumor clusters; MDSCs, myeloid-derived suppressor cells; MSI-H/dMMR, microsatellite instability-high/mismatch repair deficiency; NETs, neutrophil extracellular traps; NI, nivolumab plus ipilimumab; NLR, neutrophils-to-lymphocytes ratio; OATP1B3, organic anion-transporting polypeptide 1B3; ORR, objective response rate; OS, overall survival; PBMC, peripheral blood mononuclear cells; PD-1, programmed death protein-1; PD-L1, programmed death ligand-1; PFS, progression free survival; PIVKA-II, protein-induced by vitamin K absence or antagonist II; PLR, platelet-to-lymphocyte ratio; PPI, proton pump inhibitors; RER, relative enhancement ratio; scRNA-seq, single-cell RNA sequencing; Se, sensitivity; Sp, specificity; TAMs, tumor-associated macrophages; TANs, tumor-associated neutrophils; TCR, T-cell receptor; Teff, T-effector; TKI, tyrosine kinase inhibitors; TLS, tertiary lymphoid structures; TMB-H, tumor mutational burden-high; Tregs, regulatory T cells; VAF, variant allele frequency.
